# Rescuing Self: Transient Isolation and Autologous Transplantation of Bone Marrow Mitigates Radiation-Induced Hematopoietic Syndrome and Mortality in Mice

**DOI:** 10.3389/fimmu.2017.01180

**Published:** 2017-09-25

**Authors:** Subhajit Ghosh, Namita Indracanti, Jayadev Joshi, Prem Kumar Indraganti

**Affiliations:** ^1^Division of Radiation Biosciences, Institute of Nuclear Medicine and Allied Sciences, Delhi, India; ^2^S.N. Pradhan Centre for Neuroscience-University of Calcutta, Kolkata, India

**Keywords:** autologous bone marrow transfer, hematopoietic stem cells, homing, ionizing radiation, mitigation, hematopoietic syndrome

## Abstract

The inflamed bone marrow niche shortly after total body irradiation (TBI) is known to contribute to loss of hematopoietic stem cells in terms of their number and function. In this study, autologous bone marrow transfer (AL-BMT) was evaluated as a strategy for mitigating hematopoietic form of the acute radiation syndrome by timing the collection phase (2 h after irradiation) and reinfusion (24 h after irradiation) using mice as a model system. Collection of bone marrow (BM) cells (0.5 × 10^6^ total marrow cells) 2 h after lethal TBI rescued different subclasses of hematopoietic stem and progenitor cells (HSPCs) from the detrimental inflammatory and damaging milieu *in vivo*. Cryopreservation of collected graft and its reinfusion 24 h after TBI significantly rescued mice from lethal effects of irradiation (65% survival against 0% in TBI group on day 30th) and hematopoietic depression. Transient hypometabolic state (HMS) induced 2 h after TBI effectively preserved the functional status of HSPCs and improved hematopoietic recovery even when BM was collected 8 h after TBI. Homing studies suggested that AL-BMT yielded similar percentages for different subsets of HSPCs when compared to syngeneic bone marrow transfer. The results suggest that the timing of collection, and reinfusion of graft is crucial for the success of AL-BMT.

## Introduction

Matched allogenic bone marrow transfer (AG-BMT) has long been established and used for treating radiation-induced hematopoietic depression (1). However, the Chernobyl and other experiences showed its utility only for a fraction of victims exposed to radiation (2). Victims exposed to lower doses (<6 Gy) may not benefit from bone marrow transplantation (BMT) because at these doses, the residual hematopoietic stem cells (HSCs) would eventually cause donor marrow rejection (3, 4). Considering the limited use of AG-BMT, strategies must be developed for treating radiation-induced bone marrow (BM) aplasia and hematopoietic form of the acute radiation syndrome (hARS). Previous studies on dogs, non-human primates (NHPs), and humans have reported that if a sufficient number of HSCs could be protected (by shielding the femur), it is possible to ameliorate the myeloablative effects of chemotherapy or radiation (5–7). These studies prompted researchers to explore the efficiency of AL-BMT in the management of radiation-induced BM aplasia. This efficiency is based on the fact that quiescent HSCs survive high doses of radiation and is contributed by the heterogeneity of the absorbed dose because of the geometry of the exposure and dose attenuation related to the body’s thickness (8). The residual functional HSCs isolated soon after irradiation can be expanded and reinfused at later time points to achieve hematopoietic recovery. Contrary to AG-BMT, AL-BMT may accelerate hematopoietic recovery without causing graft-versus-host disease complications. In addition to its application in high-dose exposure patients, AL-BMT may be effective in enhancing hematopoietic recovery against moderate to sublethal radiation doses, where AG-BMT is not desired.

However, isolating a sufficient number of hematopoietic stem and progenitor cells (HSPCs) from irradiated victims remains an important concern for achieving successful engraftment and hematopoietic recovery after AL-BMT. The *ex vivo* expansion of the collected graft in the presence of antiapoptotic cytokines has been suggested to be critical because the reinfusion of the isolated BM cells without expansion results in poor engraftment and limited hematopoietic recovery (9–11). However, compromised long-term (LT) hematopoietic potential because of residual DNA damage in the isolated HSPCs remains an important concern related to these approaches (12). Moreover, the infusion of the BM several days after the radiation exposure delays effective hematopoietic recovery because BMT performed within 24 h has so far been the most effective in treating radiation-induced hematopoietic depression (13, 14). Moreover, studies on NHPs have shown that the BM collected 2 h after total body irradiation (TBI) is ideal for effective *ex vivo* expansion and engraftment (15). The BM collected at later time points resulted in the poor recovery of functional HSPCs because of a disturbed milieu and initiation of a large apoptotic burst (15). The requirement of BM collection within 2 h of radiation exposure restricts its use in cases of accidental or nuclear eventualities. The development of strategies or agents effective in preventing the loss of HSPCs both in terms of their number and functional status well beyond 2 h will increase its practical applications. Numerous investigators have reported that shortly after irradiation (from few hours to first few days), free radical-mediated oxidative stress, inflammation, and bystander effects prolong the effects on hematopoietic depression by causing the loss of cells that are not directly affected by radiation (16–18). Keeping these results in mind, in this study, we applied AL-BMT in a murine model and determined whether transient hypometabolic state (HMS) induced soon after irradiation preserves the functionality of HSPCs until 8 h after TBI.

Non-human primate models are more relevant to humans in terms of stem cell biology (19); however, they extensively restrict the detailed analysis of different aspects of AL-BMT. Mice have been used to understand the different aspects of BMT and homing (20). However, unlike large mammals, in rodents, the AL-BMT process involving a surgical procedure and postoperative care is extremely challenging and results in high mortality rates (21, 22). Moreover, irradiated mice surgically operated for BM aspiration has a risk of combined radiation injuries, which is known to accelerate radiation-induced death (23). However, in the past decade, significant advances have been made in the life saving and aseptic surgery in rodents, including mice (24). In our laboratory, we have standardized the surgical procedure and postoperative care for irradiated mice. We used mice as a model for investigating the radiomitigative efficacy of AL-BMT.

In this study, we revealed that BM collection within 2 h after TBI followed by cryopreservation for 22 h and reinfusion 24 h after TBI into the same mice rescues from the lethal effects of TBI by enhancing the hematopoietic recovery. These results suggest the efficiency of this approach, at least in the murine model, without requiring *ex vivo* graft expansion. Efforts to prolong the BM collection phase by inducing transient HMS (for a total of 8 h after TBI) have not been successful in terms of survival advantage. However, the evaluation of the BM and peripheral blood counts of the surviving animals suggest that HMS effectively preserved the functional status and number of HSPCs collected even 8 h after TBI.

## Materials and Methods

### Mouse Experiments

Animal handling and experiments were conducted in accordance with the guidelines of the Committee on the Ethics of Animals Experiments, Institute of Nuclear Medicine and Allied Sciences (INMAS), Defence Research and Development Organization, Delhi, India (Institutional Ethical Committee approval number: INM/IAEC/2013/03/04; Protocol no.: TD-10018; GO/a/99/CPCSEA). Inbred female mice (C57BL/6J) aged 10–12 weeks (weight: 25 ± 2 g) were used in this study. The animals were kept at the Experimental Animal Facility, INMAS, at an ambient temperature (*T*_a_) and relative humidity of 23–25°C and 55%, respectively. Unlimited mouse chow (Golden Feeds, Delhi, India) and tap water were provided *ad libitum*, and the animals were maintained on a 12-h light:dark cycle. All efforts were made to minimize animal number and suffering.

### Animal Irradiation

Total body irradiation was performed in partially restrained mice by using a ^60^Co γ-ray irradiator (Bhabatron-II Teletherapy Unit, Panacea Medical Technologies, Bangalore, India). For radiomitigation studies, the mice were exposed to 8.5 Gy (LD_100/30_) at a dose rate of 1 Gy/min on a rotating chamber, and the irradiation field size was 35 cm × 35 cm. The animals were partially restrained to avoid cuddling and shielding while radiation exposure; they were exposed in a group of 4. The exposure rates in the teletherapy unit were mapped by an Atomic Energy Regulatory Board-authorized health physicist by using a calibrated RadCal 0.6-cm^3^ therapy grade ion chamber/electrometer system. All irradiations were consistently provided between 12:00 and 01:00 p.m. to minimize chronosensitivity (25). The irradiated animals were monitored daily and those showing a combined score higher than 8 (posture, eye appearance, and activity scores) were humanely euthanized to minimize the pain and distress (26).

### Induction of HMS in Mice

Numerous small molecules, including adenosine receptor agonist-like adenosine monophosphate and ^6^N-cyclohexyl adenosine, are known to induce reversible HMS (27–29). By using a drug repurposing approach in conjunction with cheminformatics, the *in silico* screening of many Food and Drug Administration-approved small molecules and drug-like molecules [LOPAC 1280, JHCCL(v2), Enzo FDA library] yielded a small molecule having adenosine receptor agonist action (hereafter referred to as SJNP-1). SJNP-1, clinically used for treating depression, was used in this study to induce HMS. HMS was induced 2 h after TBI by intraperitoneally (i.p.) administering 100 mg/kg body weight (b.w.) of SJNP-1 (Sigma–Aldrich, St. Louis, MO, USA) prepared in phosphate-buffered saline (PBS). Thereafter, the animals were kept in an incubator at 15°C (for maintaining HMS) for 6 h. They were then maintained at room temperature for recovery. The core body temperature (*T*_b_) was manually measured on an hourly basis for the entire duration of the study (8 h) at different time points by using a rectal probe (RET 3, Braintree Scientific Inc., MA, USA).

### BM Aspiration from Femur, Cryopreservation, and AL-BMT

For AL-BMT, the BM from the right femur of live mice was aspirated, 2 h after TBI or 8 h after induction of HMS essentially by following a previously reported protocol, with a few modifications (24). Before surgical manipulation, 10- to 12-week-old mice were anesthetized with 80 and 10 mg/kg b.w. of ketamine and xylazine, respectively, and fur in the area where the incision was to be made was gently clipped. The entire femur and tibia were disinfected with povidone-iodine (Betadine) scrub. Thereafter, an incision was made covering the femur and tibia joint, and muscles were gently spread to clearly observe the condyle. The area was disinfected with 5% H_2_O_2_. Subsequently, by using a wet 0.5-mL syringe fit with a 27-G needle, a hole was gently drilled by turning clockwise and counter clockwise while applying pressure. The needle was inserted into the distal femur above the patella through the patellar tendon, and the BM was aspirated (~8 μL). Consistently, the aspiration was performed only once, and the tendon and overlying muscles were gently positioned and sutured to close the incision. The mice were placed on a heating pad (28°C) to recover and were returned to animal housing on complete recovery. The collected BM was washed in PBS, and the pellet was resuspended in 500 µL of cryopreserving solution [90% fetal bovine serum (FBS) and 10% dimethyl sulfoxide (DMSO)] and frozen by sequentially reducing the temperature to a final storage temperature of −80°C. The cell count and viability were assessed through flow cytometry, as previously reported (30). For AL-BMT, which was conducted at 24-h after irradiation, the cryopreserved cells were washed two times with PBS to remove traces of DMSO and serum. The final pellet was suspended in 150 µL (mean cell count: 0.5 × 10^6^ cells) of PBS and injected *via* the tail vein into the same mice. For assessing the effects of cryopreservation on the quality and quantity of different subsets of HSPCs, the BM was harvested from a separate group of untreated animals and either cryopreserved for 24 h at −80°C or freshly processed, without cryopreservation. The harvested BM was processed and stained with different surface markers for enumerating the different subsets of HSPCs. The mitochondrial membrane potential (MMP) and apoptosis were analyzed in different subsets of HSPCs.

### HSPC Enumeration and Flow Cytometry

The collected BM was treated with ice-cold 1× ammonium chloride potassium solution for 2 min at 4°C to remove red blood cells. For lineage depletion (Lin^−^), BM mononuclear cells (MNCs) were incubated with biotin-conjugated antibodies against murine CD5, Mac-1, CD45R/B220, Ter-119, and Gr-1 (Lineage Cell Depletion Kit mouse, Miltenyi Biotec GmbH, Germany). Thereafter, mature myeloid and lymphoid cells were depleted by incubating the cells with antibiotin microbeads and separating them using MS columns and MACS separator (Miltenyi Biotec GmbH, Germany), following the manufacturer’s recommendations. The Lin^−^ cell fraction was washed with PBS + 2% FBS, and the cell number was volumetrically determined through flow cytometry (30). For analyzing different HSPC populations, the Lin^−^ fraction was preincubated with anti-CD16/32 antibody (0.5 μL/10^6^ cells; Biolegend, CA, USA) to block the Fcγ receptors and then stained with anti-Sca1-PE (1 μL/10^6^ cells; Biolegend), c-Kit-APC-Cy7 (0.5 μL/10^6^ cells; Biolegend), and CD34-PerCP/Cyp5.5 (0.8 μL/10^6^ cells; Biolegend) on ice, in dark for 1 h. The frequencies of hematopoietic progenitor cells (HPCs; Lin^−^Sca1^−^c-kit^+^ cells), KSL (Lin^−^Sca1^+^c-kit^+^ cells), short-term HSCs (ST-HSCs; Lin^−^Sca1^+^c-kit^+^ CD34^+^ cells) (31), and long-term HSCs (LT-HSCs; Lin^−^Sca1^+^c-kit^+^CD34^−^ cells) (32) were analyzed using BD Accuri C6 software. Appropriate isotypes and single positive controls were also acquired whenever required for compensation, and at least 20,000 cells were acquired for each sample. The percentage of living and dead cells was determined using 7-aminoactinomycin D (7-AAD; Biolegend) exclusion procedures. For analyzing changes in the MMP in different HSPCs, after treatment with different antibodies for 45 min, rhodamine 123 (10 nM; Sigma, St. Louis, MO, USA) was added and immediately acquired. Similarly, after treating the cells with antibodies for different surface markers, they were resuspended in binding buffer containing anti-annexin V–fluorescein isothiocyanate (3 μL/10^6^ cells; Sigma) and further incubated for 15 min in the dark on ice. Thereafter, the cells were washed and acquired. The numbers of different HSPC populations were determined and presented as frequencies per million bone marrow mononuclear cells (BM-MNCs).

### Homing Studies

For carboxyfluorescein diacetate succinimidyl ester (CFSE) staining, total BM cells (0.5 × 10^6^/mL) were labeled with CFSE (5 µM) in PBS + 2% FBS for 15 min at 37°C in the dark with intermittent mixing. Labeling was terminated by adding 5 mL of ice-cold PBS + 2% FBS, and the samples were thoroughly washed to remove any unbound CFSE from the samples (33). The labeled cells (0.5 × 10^6^ cells/animal) were injected *via* the tail vein, and the animals were sacrificed 24 h after infusion (i.e., 48 h after TBI). Total BM from both femur and tibia was harvested, processed, and stained for the different subsets of HSPCs, as described earlier. CFSE-positive cells were gated, and the different subsets of HSPCs within this population were identified for assessing homing efficiencies. To assess the effects of the surgical procedure on the homing of the different subsets of HSPCs, the BM was harvested from a group of animals and cryopreserved, as described earlier. At 24 h, the cryopreserved BM samples were labeled with CFSE and infused *via* the tail vein into a group of animals exposed to TBI (a single dose of 8.5 Gy) 24 h earlier. The homing of the different subsets of HSPCs was determined by considering the number of cells infused (0.5 × 10^6^ cells) and that of cells homed onto the tibia and femur. For assessing the divisional status of the different subsets of HSPCs, the BM (0.5 × 10^6^/mL) harvested 2 h after lethal TBI was labeled with CFSE (20 µM), as mentioned earlier and was reinfused into the same animals. The animals were sacrificed 96 h after TBI, and the BM was harvested, depleted of RBC and stained for different surface antigens, as mentioned earlier. The samples were acquired through flow cytometry. For undiluted CFSE-labeled cells *in vivo*, the BM harvested from untreated animals were labeled with CFSE and infused into the recipient mice. Subsequently, 2 h later, the animals were sacrificed, blood was collected by cardiac puncture, the total mononuclear fraction was harvested, and samples were acquired through flow cytometry. The mean fluorescence of CFSE-positive cells was considered as undiluted samples.

### Radiomitigation—30-Day Survival Assay

After performing AL-BMT, the animals were housed and maintained at the experimental animal facility. All animals received ciprofloxacin (45 mg/kg b.w./day; i.p.) for three consecutive days after bone marrow transfer. The kinetics of the overall survival, defined as the time from the date of irradiation to the date of death of the examined mice, was analyzed using the (two-tailed) log-rank test. The 30-day survival rate was compared between the autologous transfer and radiation-only control groups.

### Cytological and Histological Evaluation of BM

To determine the effects of AL-BMT on recovery from hematopoietic depression, BM cellularity was evaluated in BM smears of the surviving animals. For the irradiation alone group, BM smears were prepared from the animals having a combined distress score of more than 8 (the dying animals; on the 13th day). These smears were prepared using a paint brush and stained with May–Grünwald–Giemsa stain. For histology, femurs from different groups of animals were dehydrated and processed following routine procedures and stained with hematoxylin and eosin (H&E). Hematological profiling was performed within 1 h of collection of blood by using an automatic hematology analyzer (Celltac α, Nihon Kohden, Japan).

## Results

### Rescuing Self: BM Aspirated from Lethally Irradiated Animals within 2 h and Readministered after 24 h Can Rescue Animals from Irradiation-Induced Lethality

**Figure 1:**
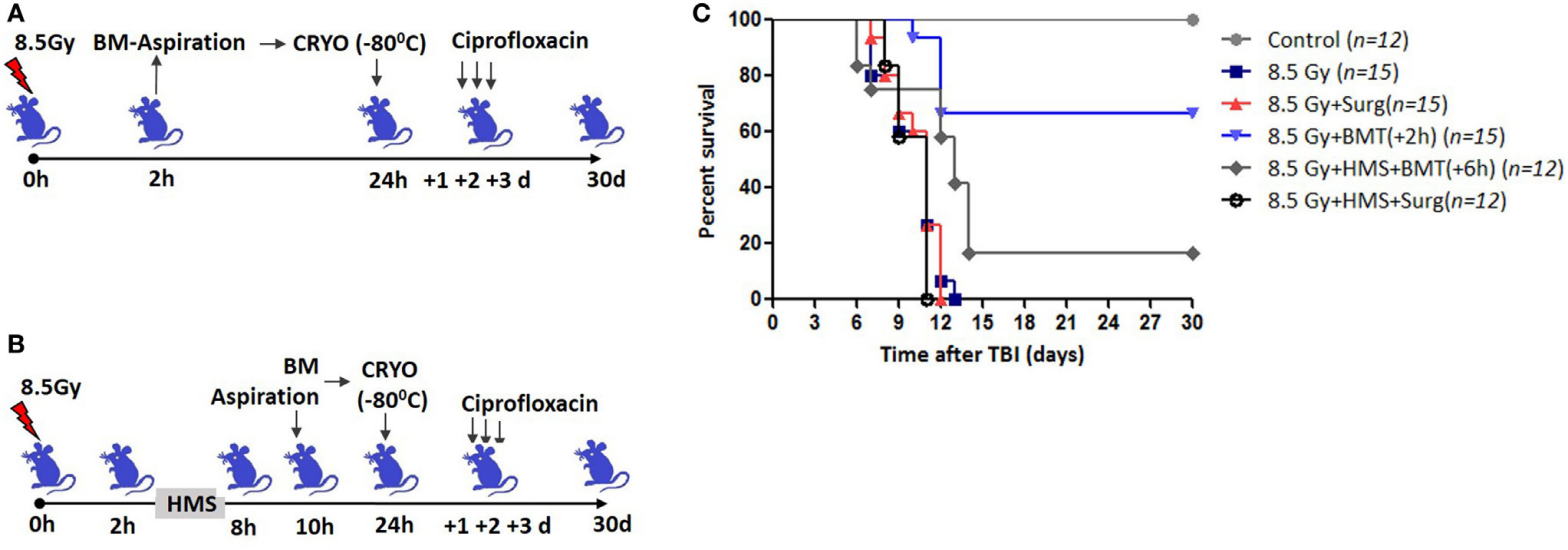
Bone marrow collected within 2 h after total body irradiation (TBI) rescues mice from lethal effects when reinfused 24 h later. **(A)** Schema of autologous bone marrow transplantation setup. Bone marrow was collected 2 h after TBI (8.5 Gy), cryopreserved for 22 h, and reinfused 24 h after TBI. **(B)**. 2 h after TBI, SJNP-1 was administered through i.p. route, and mice were kept at an ambient temperature (*T*_a_) of 15°C for 6 h. Thereafter the mice were shifted to *T*_a_ (25°C) for recovery and 2 h later [that is, 8 h after hypometabolic state (HMS)] bone marrow was collected, cryopreserved, and reinfused 24 h after TBI. **(C)**. Kaplan–Meier plot showing survival rates of mice with different treatments.

**Figure 2:**
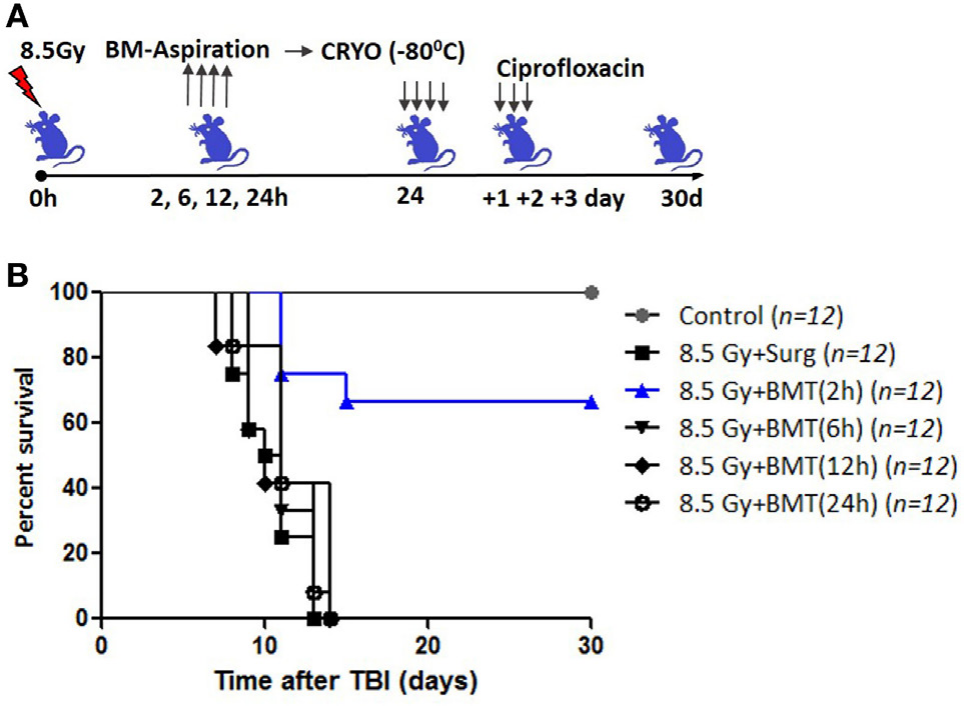
Effect of bone marrow collection time on radiomitigative effect of AL-BMT. **(A)** Schema of autologous bone marrow transplantation. Bone marrow was collected at indicated time points after total body irradiation (TBI) (8.5 Gy), cryopreserved and reinfused at 24 h after TBI. **(B)**. Kaplan–Meier plot showing survival rates of mice with different treatments.

Total body irradiation (8.5 Gy; dose rate: 1.0 Gy/min) resulted in 100% lethality in 30 days (mean survival time: 12 days; Figures 1A,C). The surgical procedure performed on irradiation control mice, to assess the effects of irradiation on survival, did not result in any changes in radiation-induced mortality (data not shown). The mice that received the cryopreserved unprocessed total BM (0.5 × 10^6^ nucleated cells/mice), 24 h after TBI, showed 65% survival advantage after 30 days compared to 0 survival in the 8.5 Gy irradiation and surgically manipulated group (BM aspiration procedure; hereafter referred as TBI + Surg), which received a vehicle. To assess the efficiency of transient HMS on extending the duration before the BM collection phase, the effect of SJNP-1 was investigated (Figures 1B,C). The administration of a single dose of SJNP-1 (100 mg/kg b.w.) 2 h after TBI followed by placing them at a *T*_a_ of 15°C resulted in a gradual reduction in *T*_b_ gradually and nadir (~32°C) reached 2 h after administration (Figure S1 in Supplementary Material). The reduced *T*_b_ remained consistent throughout the experiment (6 h). The *T*_b_ of the animals quickly returned to normal levels within 2 h of shifting from 15°C to room temperature. Furthermore, the induction of HMS 2 h after TBI followed by BM aspiration after 8 h under general anesthesia did not induce any immediate death and showed a mortality trend similar to that observed in the TBI alone or TBI + Surg group. However, the AL-BMT of aspirated BM (collected 8 h after HMS) reinfused 24 h after TBI resulted in a survival advantage (16%) in comparison to the 0 survival observed in the TBI + HMS + Surg group (Figure 2). Thereafter, to determine the optimal time point, AL-BMT experiments were performed using the BM collected at different time points after TBI (Figure 2). The results indicated that the AL-BMT of BM grafts collected at different “delayed” time points after TBI (6, 12, or 24 h after TBI) did not exhibit any mitigative effects, and all animals succumbed to TBI-induced lethality (Figure 2B).

### AL-BMT Ameliorates Irradiation-Induced Leukocytopenia, Erythropenia, and Thrombocytopenia

**Figure 3:**
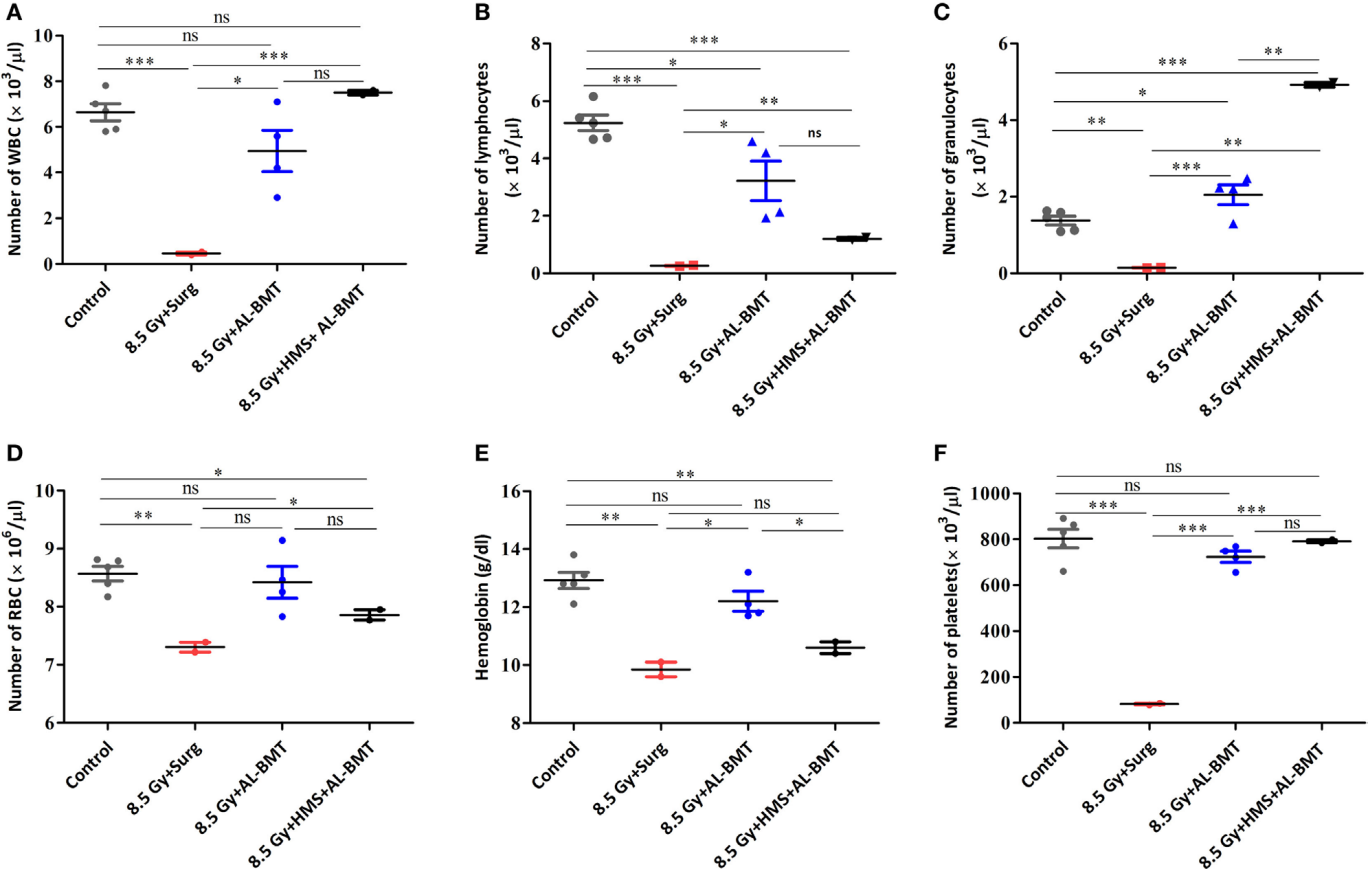
Hematopoietic parameters of recipient mice transplanted with autologous bone marrow collected 2 h after lethal irradiation or 8 h after induction of hypometabolic state (HMS). Changes in the number of white blood cell (WBC) **(A)**, lymphocytes **(B)**, granulocytes **(C)**, RBC **(D)**, hemoglobin **(E)**, and platelets **(F)** measured on 30th postirradiation day. Each value is a mean ± SEM (*n* = 4–6 animals/group), and comparisons were done as indicated using unpaired *t*-test (*p < 0.05, **p < 0.01, ***p < 0.001, ns, not significant).

After demonstrating the radiomitigative potential, we determined the effects of AL-BMT on recovery from radiation-induced leukocytopenia, erythropenia, and thrombocytopenia (Figure 3). TBI caused a significant loss (93 compared with 0% loss in the untreated control group) of white blood cells (WBCs) on day 13. However, AL-BMT significantly mitigated radiation-induced loss of WBC (25%; Figure 3A). The TBI + HMS + Surg group showed a similar trend of leukocytopenia and erythropenia and a decline in thrombocytes, as also shown by the TBI + Surg and TBI alone groups (data not included). We consistently observed mice death during the night, which complicated blood collection from a large number of samples. Similarly, the surviving animals (16%), which received the BM collected 8 h after HMS induction, showed effective recovery compared with those in the TBI + Surg group, which received a vehicle, at 24 h, in place of BM (Figure 3A).

We evaluated two major subsets of WBCs, which play a significant role in establishing a competent immune status and facilitating recovery from hARS: lymphocytes and granulocytes (Figures 3B,C). In the untreated control group, lymphocytes and granulocytes accounted for 78 and 22% of total WBCs, respectively. The TBI + Surg group revealed a significant loss of both lymphocytes and granulocytes compared with the vehicle-treated un-irradiation control group (Figures 3B,C). Moreover, a slight myeloid bias (M-bias) with WBCs constituting 60 and 40% of lymphocytes and granulocytes, respectively, was evident. The animals that underwent AL-BMT also showed a slight M-bias with the lymphoid (57%) to myeloid (42%) ratio. However, the surviving animals (16%) that received the BM collected at 8 h after HMS induction showed a strong myeloid shift with WBCs comprising 16 and 84% of lymphocytes and granulocytes, respectively. As was observed with WBC, the RBCs and the total hemoglobin content have also exhibited a similar trend (Figures 3D,E). Similarly, the TBI + Surg group showed a significantly reduced (90 ± 1.5% loss) platelet count compared with the vehicle-treated un-irradiation control group (Figure 3F). AL-BMT effectively recovered the platelet count (10% loss compared with 90% loss in the IR group) on day 30 as compared with the count in the vehicle-treated un-irradiation control group. Similar to WBCs, the animals that received AL-BMT by using the BM collected hours after HMS induction, showed complete recovery; the number of platelets was found similar in this group and the untreated control group (Figure 3F).

Recovery from radiation-induced hARS depends on the number of surviving and functional HSCs in the BM, which undergoes division and forms multilineage progenitors that further differentiate into varied lineages. After demonstrating the improved recovery of the different subsets of hematopoietic cells in peripheral blood, the cellular status of the BM was assessed (Figures S3A–L in Supplementary Material). The BM smears prepared on days 30 and 14 from the mice that received AL-BMT 24 h after TBI or those that received a vehicle (24 h after TBI + Surg) respectively, were examined. In corroboration with the decreased peripheral blood cell count, TBI induced a significant loss of BM cellularity with sparse pockets of nucleated hematopoietic cells (Figures S3C,D in Supplementary Material). However, the animals that received AL-BMT showed significant recovery with apparently higher numbers of nucleated hematopoietic cells with active trilineage hematopoiesis compared with that of the TBI + Surg mice, albeit less than that of the untreated control mice (Figures S3E,F in Supplementary Material). As observed with peripheral blood counts, the surviving animals (16%) that received the BM collected 8 h after HMS induction revealed BM cellularity almost equal to that in the normal untreated control group (Figures S3G,H in Supplementary Material).

The evaluation of histopathological changes in longitudinally sectioned and H&E-stained femurs in the vehicle-treated mice showed that TBI caused aplasia in the surviving animals with a few islands of nucleated hematopoietic cells and massive deposition of adipose cells on day 14 (Figure S3J in Supplementary Material). The animals that received the BM collected 2 h after TBI showed significant recovery and increased cellularity (Figure S3K in Supplementary Material). While the animals that received BM grafts collected 8 h after HMS induction revealed the highest recovery from irradiation-induced aplasia and adipogenesis (Figure S3L in Supplementary Material).

### Isolation and Cryopreservation Effectively Preserves Different Subsets of HSPCs

The number of functional HSPCs in the aspirated BM samples is critical for achieving successful engraftment and hematopoietic recovery; hence, this number in aspirated grafts was determined. The procedure involved the collection of BM cells followed by cryopreservation for 22 h before reinfusion. Therefore, assessing the effects of the procedure on the viability and number of the different subsets of HSPCs was important. The freezing method used in this study resulted in a significant loss of viability (91% of cells were viable, as determined using the 7-AAD uptake assay) in BM-MNCs compared with freshly processed BM samples (97% viable) (Figure S2 in Supplementary Material). However, TBI did not aggravate the effects of cryopreservation and did not result in any further increase in the number of dead cells when harvested 2 h after TBI (90% of cells were viable compared with 91% for the BM aspirated from the un-irradiation control animals and processed similarly). Subsequently, the effects of cryopreservation on the loss of different subsets of HSPC were evaluated (Figure S4 in Supplementary Material). The cryopreservation of the BM aspirated from the vehicle-treated un-irradiated animals for 22 h resulted in a significant loss of HPCs and LT-HSCs compared with that of freshly processed BM samples obtained from vehicle-treated un-irradiated animals (Figures S4B,E in Supplementary Material). However, both KSL and ST-HSC populations remained unaffected (Figures S4C,D in Supplementary Material). TBI resulted in a significant loss of HPCs and LT-HSCs, whereas KSL and ST-HSCs were not significantly affected as compared to the BM of the vehicle-treated un-irradiated animals collected and processed immediately without cryopreservation (Figures S4B–E in Supplementary Material). The BM samples harvested 2 h after TBI did not exhibit any significant changes in the number of different subsets of HSPCs and yielded results similar to those obtained in the vehicle-treated un-irradiated group (data not shown).

Dimethyl sulfoxide and cryopreservation result in a significant loss of the clonogenicity of HSPCs (34, 35). To assess the functional integrity of the surviving HSPCs, changes in the MMP and apoptosis were evaluated in the different subsets of HSPCs (Figures S5 and S6 in Supplementary Material). The BM of the vehicle-treated un-irradiated animals harvested and processed immediately revealed that 59, 55, 58, or 48% of HPCs, KSL cells, ST-HSCs, and LT-HSCs possessed highly polarized mitochondria (high rhodamine 123 intensity; Figures S5B–E in Supplementary Material). The cryopreservation procedure used in this study did not affect the percentage of different HSPCs with highly polarized mitochondria compared with the BM of the vehicle-treated un-irradiated animals harvested and processed immediately (Figures S5B–E in Supplementary Material). Moreover, TBI did not influence the MMP of different HSPCs when compared with their corresponding cryopreserved HSPCs from the vehicle-treated un-irradiated animals (Figures S5B–E in Supplementary Material). However, LT-HSCs were an exception, and TBI significantly reduced the fraction of LT-HSCs with highly polarized mitochondria (14%) compared with both freshly processed and cryopreserved BM samples harvested from the vehicle-treated un-irradiated animals (Figure S5E in Supplementary Material). In comparison with the freshly processed HSPCs obtained from the vehicle-treated un-irradiated animals, TBI reduced the frequency of cells with highly polarized mitochondria in different HSPCs, except in HPCs (Figures S5B–E in Supplementary Material).

Previously, studies have investigated the effects of cryopreservation, a known inducer of apoptosis, in various cell types (36–38). In concordance with the previous reports, cryopreservation induced significant increases in apoptosis levels in different subsets of HSPCs (Figures S6B–D in Supplementary Material). However, LT-HSCs exhibited almost complete resistance to cryopreservation-induced apoptosis (Figure S6E in Supplementary Material). Moreover, the cryopreservation of the BM samples collected 2 h after TBI exhibited an increase in the apoptotic fraction in the different subsets of HSPCs compared with freshly processed BM samples from the vehicle-treated un-irradiated animals (Figures S6B–E in Supplementary Material). However, the changes were not significant compared to the BM harvested from the vehicle-treated un-irradiated animals and then cryopreserved (Figures S6B–E in Supplementary Material).

**Figure 4:**
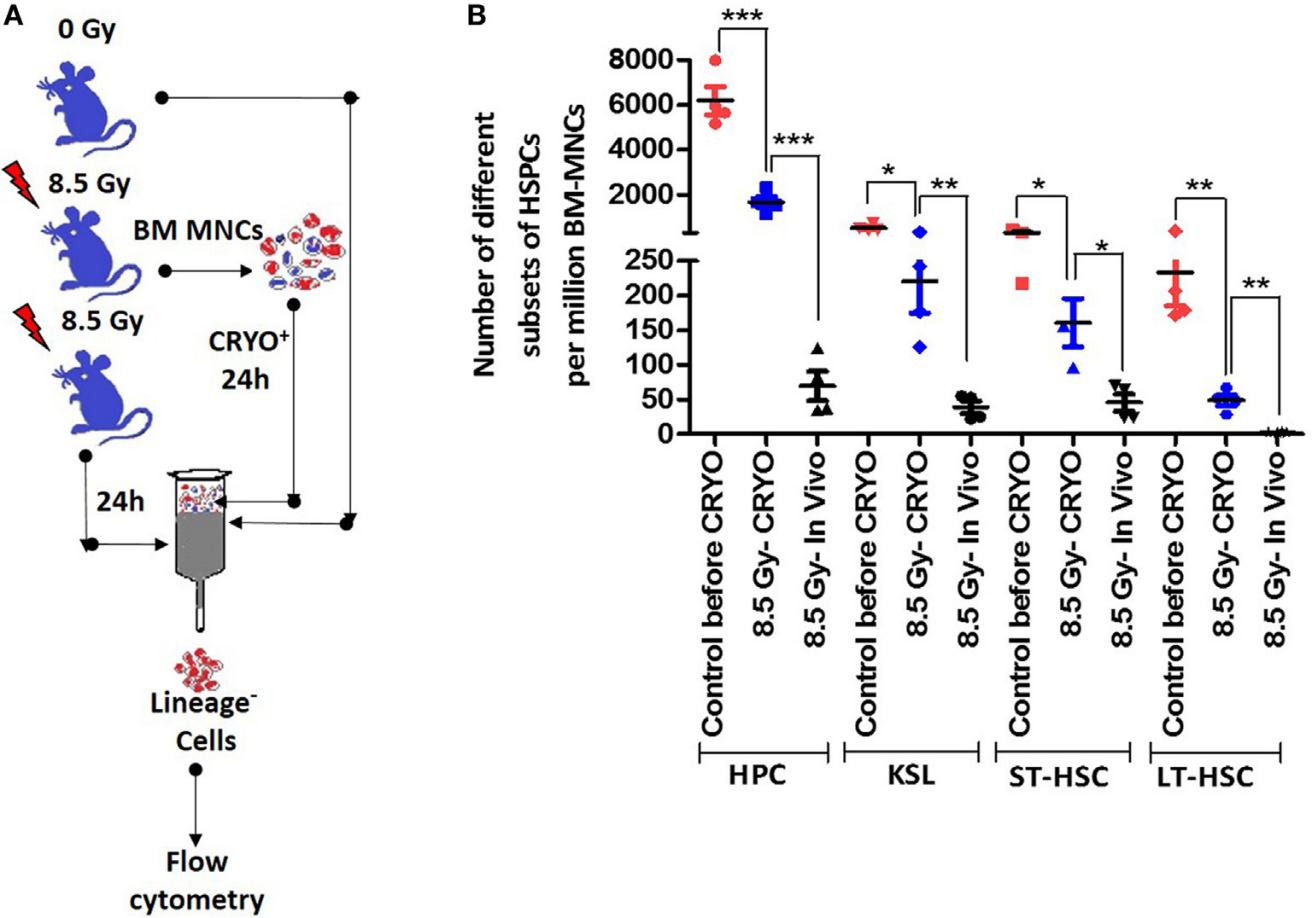
Cryopreservation procedure preserves different hematopoietic stem and progenitor cells (HSPCs) better than in irradiated animals *in vivo*. **(A)** Schema of experimental setup. **(B)** Bone marrow was aspirated 2 h after lethal total body irradiation (TBI), cryopreserved for 22 h followed by processing and enumeration of different subsets of HSPCs. Bone marrow collected 24 h after TBI or from vehicle-treated un-irradiated animals were used for comparison. The fractions of different HSPCs that are annexin V positive were deducted from their total number and used for comparisons. Each value is a mean ± SEM (*n* = 4–6 animals/group), and comparisons, as indicated, were done for statistical significance using unpaired *t*-test. *p < 0.05, **p < 0.01, ***p < 0.001.

To ascertain that the timely removal of BM cells circumvents the excessive loss of HSPCs because of a disturbed BM milieu, the number of surviving and functional HSPCs in aspirated and cryopreserved BM samples *vis-à-vis* that of different HSPCs present 24 h after irradiation *in vivo* was determined. The aspiration of BM samples 2 h after TBI followed by cryopreservation resulted in a statistically significant preservation of the different subsets of HSPCs compared with the number of HSPCs present 24 h later in the BM of TBI-exposed animals (Figures 4A,B). However, compared with freshly processed BM harvested from the vehicle-treated un-irradiation control animals, the number of different HSPCs were significantly low (Figure 4B).

### Surgical Stress Reduces the Homing of Different Subsets of HSPCs both in AL-BMT and SG-BMT Setups

**Figure 5:**
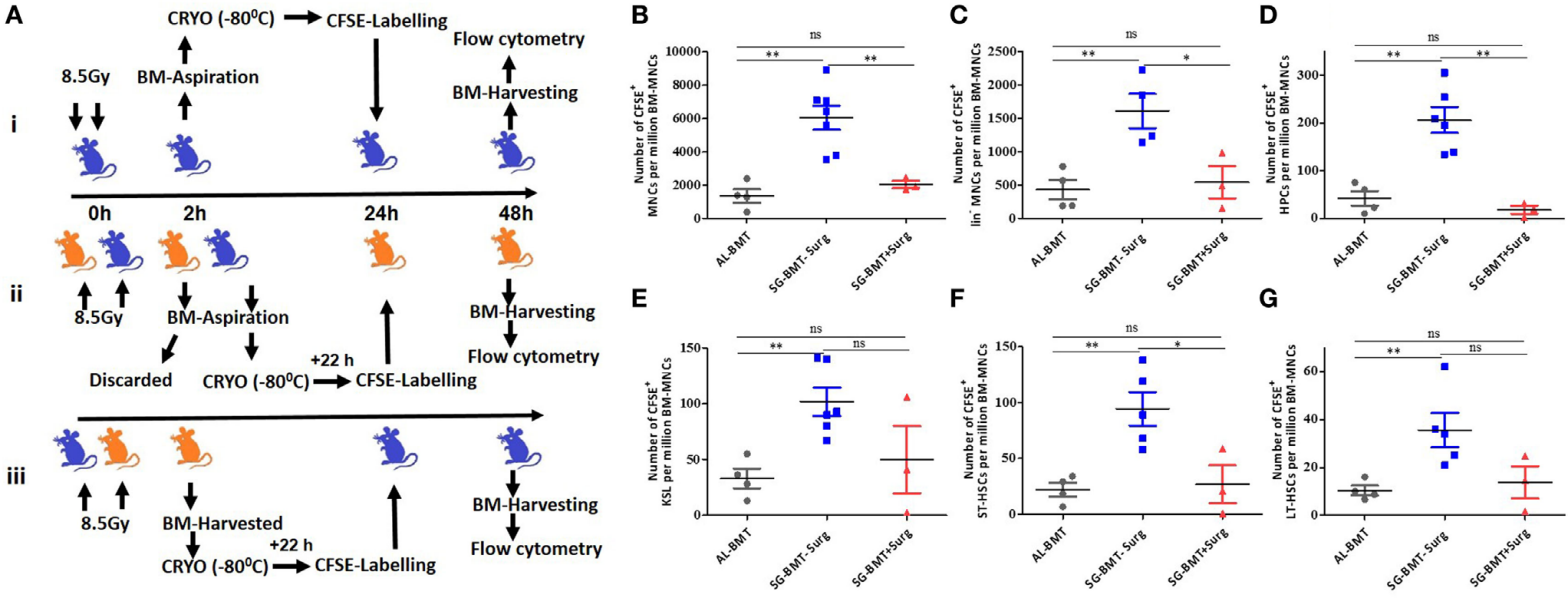
Homing of different hematopoietic stem and progenitor cells (HSPCs) in autologous and syngeneic BMT setup. **(A)** Schema of experimental setup for evaluating homing of different HSPCs in case of AL-BMT (**i)**, SG-BMT with surgery (**ii**), and SG-BMT without surgery (**iii**) setup. **(B–G)** Number of different subsets of HSPCs homed into femur 24 h after transplantation of indicated grafts with or without surgical manipulations. Each value is a mean ± SEM (*n* = 4 animals/group), and comparisons, as indicated, were done for statistical significance using unpaired Student’s *t*-test (*n* = 4; *p < 0.05, **p < 0.01). Surg, surgery.

Successful engraftment depends on the number of HSPCs homed in the BM niche, and the performance of small grafts can be improved by enhancing their homing potential (39, 40). A study suggested that AG-BMT and SG-BMT have a similar homing potential (41). After demonstrating the survival advantage of AL-BMT in mice, the homing potential of different subsets of HSPCs was evaluated in a similar setup. Numerous factors, including stress, influence the homing of stem cells and successful outcomes of bone marrow transfer (42, 43). Therefore, the effects of the surgical procedure under anesthesia on the homing of HSPCs were investigated (Figure 5A). Surgical stress affected the homing of CFSE-labeled BM-MNCs, the Lin^−^ fraction, and the different subsets of HSPCs in the tibia and femur at 24 h after BM transfer compared with syngeneic transplantation performed without surgical manipulations (Figures 5B–G). Moreover, the transfer of autologous CFSE-labeled BM cells revealed reduced homing in the tibia and femur compared with SG transfer performed without surgery (Figures 5B–G). SG-BMT involving a surgical procedure and AL-BMT revealed similar homing potential (Figures 5B–G). Consistent with these homing results, evaluation of radiomitigative action of using survival as an end point revealed that both SG-BMT (with surgery) and AL-BMT groups have shown similar radiomitigative action (Figure S8 in Supplementary Material). While, SG-BMT done in a setup without the involvement of surgical procedure resulted in an increased survival clearly suggesting the negative impact of surgical procedure (Figure S8 in Supplementary Material).

**Figure 6:**
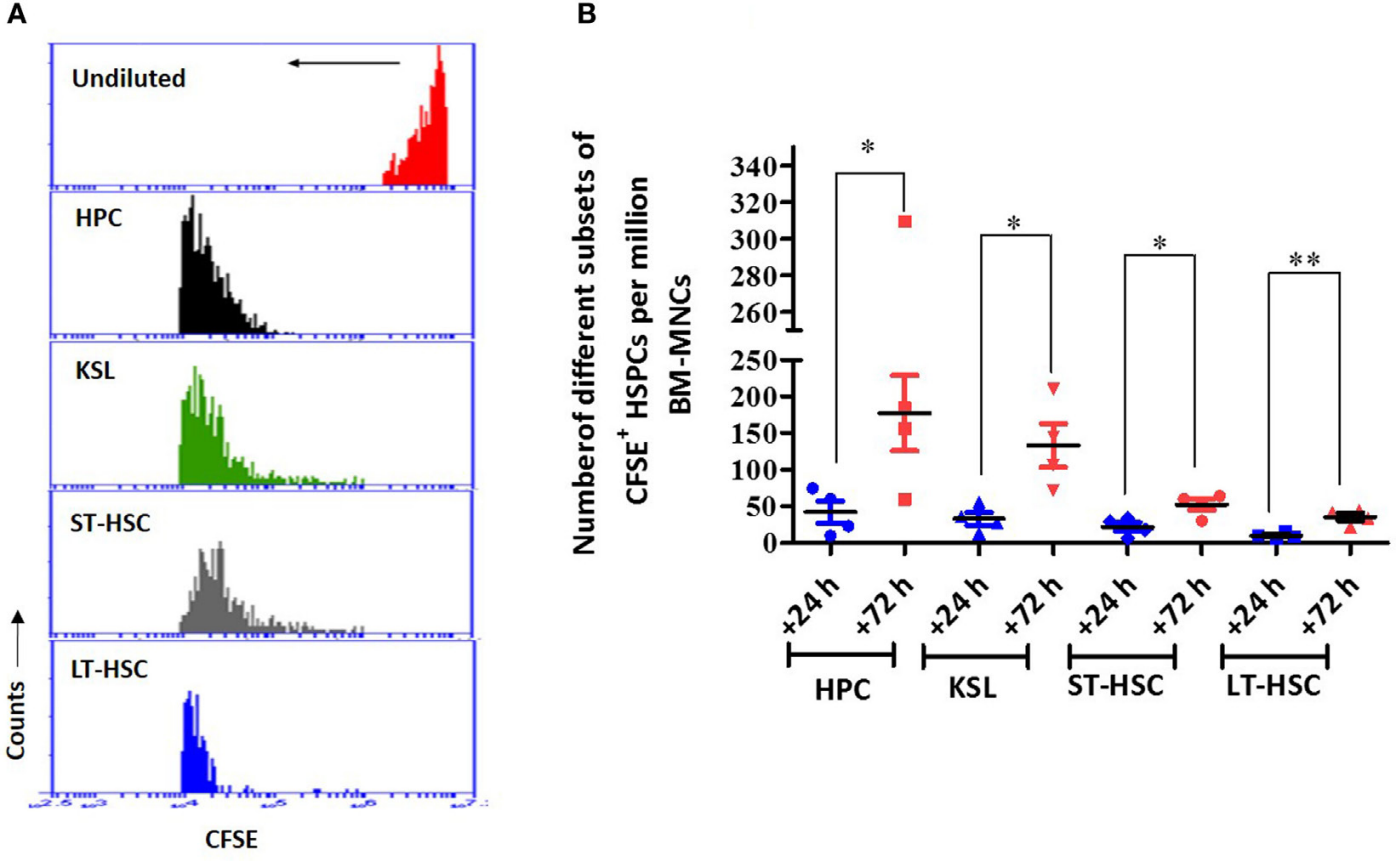
The proliferation status of transplanted bone marrow cells that have homed into bone marrow. **(A)** Representative example of carboxyfluorescein diacetate succinimidyl ester (CFSE) dilution assay. Bone marrow was harvested 72 h after transplantation, and CFSE^+^ cells were gated and mean fluorescence of the population was analyzed for CFSE dilution. Undivided bone marrow cells (red), hematopoietic progenitor cells (HPCs) (black), KSL (green), short-term hematopoietic stem cells (ST-HSCs) (gray), and long-term hematopoietic stem cells (LT-HSCs) (blue) (*n* = 4). **(B)** Enumeration of different subsets of CFSE^+^ hematopoietic stem and progenitor cells (HSPCs) in bone marrow harvested 72 h after AL-BMT. Each value is a mean ± SEM (*n* = 4 animals/group), and comparisons, as indicated, were done for statistical significance using unpaired Student’s *t*-test (*n* = 4; *p < 0.05, **p < 0.01).

To determine whether the transplanted autologous BM contributed to the overall postirradiation hematopoietic recovery, the dilution of CFSE fluorescence, indicating the divisional history of cells, was assessed 24 h after transfer (Figure 6). The CFSE dilution assay clearly indicated that among the different HSPCs analyzed in this study, all HPCs underwent division, as evident from the single peak without any shoulder (Figure 6A). Similarly, KSL, ST-HSC, and LT-HSC populations revealed significantly diluted CFSE fluorescence (a shift toward the left), but a significant shoulder was evident, indicating heterogeneity in the number of divisions of the cells (Figure 6A). The enumeration of the CFSE-positive HSPCs in the BM harvested 72 h after AL-BMT (96 h after TBI) indicated that the number of HPCs, KSL cells, ST-HSCs, and LT-HSCs significantly increased compared with that of the corresponding subsets harvested at 24 h after BM transfer (Figure 6B).

## Discussion

AL-BMT, despite its limitations, merits attention as an effective option for managing hARS in patients overexposed to radiation. Considering the relevance of AL-BMT, several researchers have proposed preserving the BM of nuclear power plant workers and first responders who are at the highest risk of radiation overexposure (44). However, any such efforts would rarely be cost-effective and may not be a feasible option for underdeveloped and developing countries. Advancements made in the past decade in the field of BM collection (45) and cryopreservation have warranted investigating AL-BMT as an effective strategy for managing hARS. The radiomitigative action of AL-BMT, demonstrated in this study, can be attributed to the transient isolation of residual HSC (which survived from radiation effects) from inflamed BM environment and protracted oxidative stress until the BM niche becomes conducive for engrafting and reconstitution. We supported this assumption by demonstrating that the isolation and cryopreservation of BM grafts more efficiently preserves different HSPCs than those of the BM of an irradiated animal, at least during the first 24 h after lethal TBI. This result is in concordance with a recent report by Ishida et al (32) who showed that elevated tumor necrosis factor alpha (TNF-α) levels in inflamed BM contribute to the compromised reconstitution of donor BM. However, such a direct comparison between *in vivo* and a vial containing BM frozen to −80°C is complex considering the number of influencing variables involved. The results could be skewed because of the mobilization of the residual HSPCs to extramedullary hematopoietic sites, which led to a reduced number of critical HSPCs in the BM collected 24 h after irradiation (46). The time point selected for reinfusing the cryopreserved BM was based on a time course study of radiation-induced proinflammatory cytokines, including TNF-α, which peaked after 2–4 h and became normal by 24 h (47–49). Notably, the reconstituted hematopoiesis post AL-BMT showed a shift toward the myeloid lineage, and the M-bias was strongly evident in the mice that received the BM collected 8 h after the induction of hypometabolism. A recent study suggested that the transfer of LT-HSCs pretreated with an antioxidant, *N*-acetyl cysteine, led to myeloid reconstitution (32). The M-bias observed in this study could partially be explained by considering that SJNP-1, which was used for inducing HMS, is an effective free radical scavenger and antioxidant. It must be validated whether the treatment of HSCs with antioxidants leads to a selective protection or proliferation of the M-bias HSPCs. In this study, ciprofloxacin was administered as a part of supportive care after surgical manipulation. Antibiotics are typically known to offer radioprotection, raising question about their contribution to overall radiomitigative action (50). However, the contribution of three suboptimal doses of ciprofloxacin can be ruled out because it was reported to be an ineffective mitigator (50).

In this study, we used a clinically established approach, reversible hypothermia or HMS, for preserving the BM *in vivo* and as a possible strategy for extending the collection phase after TBI. The induction of the HMS state, followed by the collection of grafts under general anesthesia resulted in reduced survival advantage, which could be attributed to the inability of the rodent model to survive the prolonged period of stress. This assumption is in accordance with previous reports that the prolonged maintenance of animals in HMS increases mortality (27). However, considering the efficient hematopoietic recovery observed in the residual animals, validating the usefulness of reversible HMS in larger mammals is important. In this study, we observed a significant survival advantage with 0.43% (5 × 10^5^ cells) of the BM grafts (assuming tibia and femurs constitute 25% of total BM in C57 Bl/6J mice) (51) collected and reinfused without further refinement. This result is in agreement with previous reports showing that 2.5 × 10^5^ BM nucleated cells per mice can render protection against lethal doses of ionizing radiation (52, 53). However, because cryopreservation significantly induced early apoptotic events (positive for annexin V staining) and by assuming that the apoptotic fraction will be eventually eliminated *in vivo*, on an average the mice have received ~830, 110, 80, and 25 HPC, KSL, ST-HSC, and LT-HSC, respectively, which in an ideal scenario should be able to reconstitute hematopoiesis and radioprotect mice (31). However, they rarely engraft with absolute efficiencies, and typically, a much higher number of KSL cells (2,000 cells) or LT-HSCs (50 KSL CD34^−^ cells) in isolation are required for achieving radioprotection (54). Moreover, we showed that the surgical procedure significantly reduced the homing of different HSPCs, further complicating the situation. The protective effect observed in this study could be attributed to the use of total BM containing different committed progenitors and the most primitive HSCs. The committed progenitors (ST-HSCs and HPCs) facilitate the initial engraftment and LT-HSCs ensure durable engraftment.

The CFSE dilution assay, an effective method for investigating the division of the transplanted cells, clearly suggested that the transplanted BM cells are functional and dividing, which must have contributed to the hematopoietic recovery. This is consistent with previous observations that 2 × 10^5^ of total BM [considering that KSL cells account for 0.1% (nearly 200 KSL cells) of BM nucleated cells] can protect mice from radiation-induced mortality (53). However, the duration of this study enabled the assessment of ST reconstitution only, and the effect of AL-BMT on LT reconstitution must be investigated. Moreover, a relatively lower radiation dose was used in this study, which probably requires a lesser number of HSPCs compared with 2,000 KSL cells or 2 × 10^5^ BM cells containing 200 KSL cells for conferring protection against supralethal doses (9.8 Gy). Notably, unlike anti-apoptotic cytokine-treated BM, which raises concerns about the persisting mutations and compromised LT repopulation (12), the untreated BM used in this study can be expected to effectively perform LT repopulation. However, assessing the persisting DNA damage by using markers, such as gamma-H2AX, or chromosomal aberrations in transplanted cells can be informative.

Bone marrow mononuclear cells are established as a source of mesenchymal stem cells (MSCs) and when cotransplanted, they enhance the engraftment of HSPCs (55–57). In this study, we used total BM without further processing, and enumeration studies revealed 0.3% of MSCs in the samples (data not shown). Although the number of MSCs was much lower than the minimum threshold (58) and MSCs were unlikely to affect the hematopoietic recovery solely by themselves, their contribution to HSPC engraftment cannot be ruled out. Studies conducted using a baboon model have reported that the cografting of MSCs improves the hematopoietic potential of small grafts (9).

In conclusion, this study proposes that AL-BMT is a viable strategy and that the timing of collection and reinfusion can alleviate the requirement of *ex vivo* expansion. The efficiency of AL-BMT in cases of radiation overexposure must be assessed. However, we suggest that people with early symptoms of large total absorbed doses (exposures of more than 4 Gy) typically present with vomiting within 1 h (59) and may be triaged to undergo BM collection and cryopreservation. Studies must validate reversible HMS as a strategy for prolonging the BM collection phase, which would markedly improve the clinical utility of AL-BMT.

## Ethics Statement

Animal handling and experiments with mice were carried out in accordance with the approval from, the Committee on the Ethics of Animals Experiments, Institute of Nuclear Medicine and Allied Sciences (INMAS), Defence Research and Development Organization (DRDO), Delhi, India (Institutional Ethical committee number under which this study has been approved is INM/IAEC/2013/03/; Protocol no.: TD-10018; GO/a/99/CPCSEA). All experiments were performed following the protocols approved by the Committee on the Ethics of Animal Experiments of INMAS, Delhi, India.

## Author Contributions

PI, SG, and NI conceived and designed the experiments; analyzed the data; and wrote the main manuscript. SG, NI, and JJ performed the experiments. All the authors reviewed the manuscript.

## Conflict of Interest Statement

The authors declare that the research was conducted in the absence of any commercial or financial relationships that could be construed as a potential conflict of interest.

## Abbreviations

AG-BMT, allogenic bone marrow transplantation; AL-BMT, autologous bone marrow transplantation; BM-MNC, bone marrow mononuclear cells; BMT, bone marrow transplantation; CFSE, carboxyfluorescein diacetate succinimidyl ester; CHA, ^6^N-cyclohexyl adenosine; hARS, hematopoietic form of the acute radiation syndrome; HMS, hypometabolic state; HPC, hematopoietic progenitor cell; HSC, hematopoietic stem cell; HSPCs, hematopoietic stem and progenitor cells; KSL, Lin^−^Sca1^+^c-kit^+^ cells; LT-HSCs, long-term hematopoietic stem cells; MMP, mitochondrial membrane potential; MSCs, mesenchymal stem cells; NHP, non-human primates; SJNP-1, code name of a small molecule with adenosine receptor agonist action; ST-HSCs, short-term hematopoietic stem cells; *T*_a_, ambient temperature; TBI, total body irradiation.
