# Supplementary material for: Rescuing Self: Transient Isolation and Autologous Transplantation of Bone Marrow Mitigates Radiation-Induced Hematopoietic Syndrome and Mortality in Mice
Source: Front Immunol. 2017 Sep 25;8:1180. doi: 10.3389/fimmu.2017.01180 (PMC5622201; doi:10.3389/fimmu.2017.01180)
Supplement: Supplementary file 1 [file Data_Sheet_1.doc]

**Supporting Information**

Rescuing self: Transient isolation and autologous transplantation of bone marrow mitigates radiation induced hematopoietic syndrome and mortality in mice

Subhajit Ghosh^1&2^, Namita Indracanti^1^, Jayadev Joshi^1&2^, Prem Kumar Indraganti^1*^

^1^Division of Radiation Biosciences,

Institute of Nuclear Medicine and Allied Sciences,

Brig SK Majumdar Road, Timarpur, Delhi-INDIA

^2^S.N.Pradhan Centre for Neuroscience-University of Calcutta, Kolkata-INDIA

*Correspondence and requests for materials should be addressed:

Dr. Prem Kumar Indraganti email: [prem_indra@yahoo.co.in](mailto:prem_indra@yahoo.co.in)

**Supplementary Figure S1.** Changes in core body temperature of mice administered with 100 mg/kg b.w. of SJNP-1 and placed at Ta (15 ± 2 °C) for a duration of 6 hours. The bottom line is the ambient temperature recorded for the duration of the experiment. Each value is a mean ± SEM (n=7 animals/group).

**Supplementary Figure S2**. Cryopreservation procedure results in loss of viability. The total bone marrow samples from different groups were depleted of RBC and either processed for assessing the viability immediately or frozen for 22 hours before assessing. Each value is a mean ± SEM (n=at least 6 animals/group) and comparisons, as indicated, were done for statistical significance using unpaired student t test. (n=4). ***p< 0.001, ns not significant.


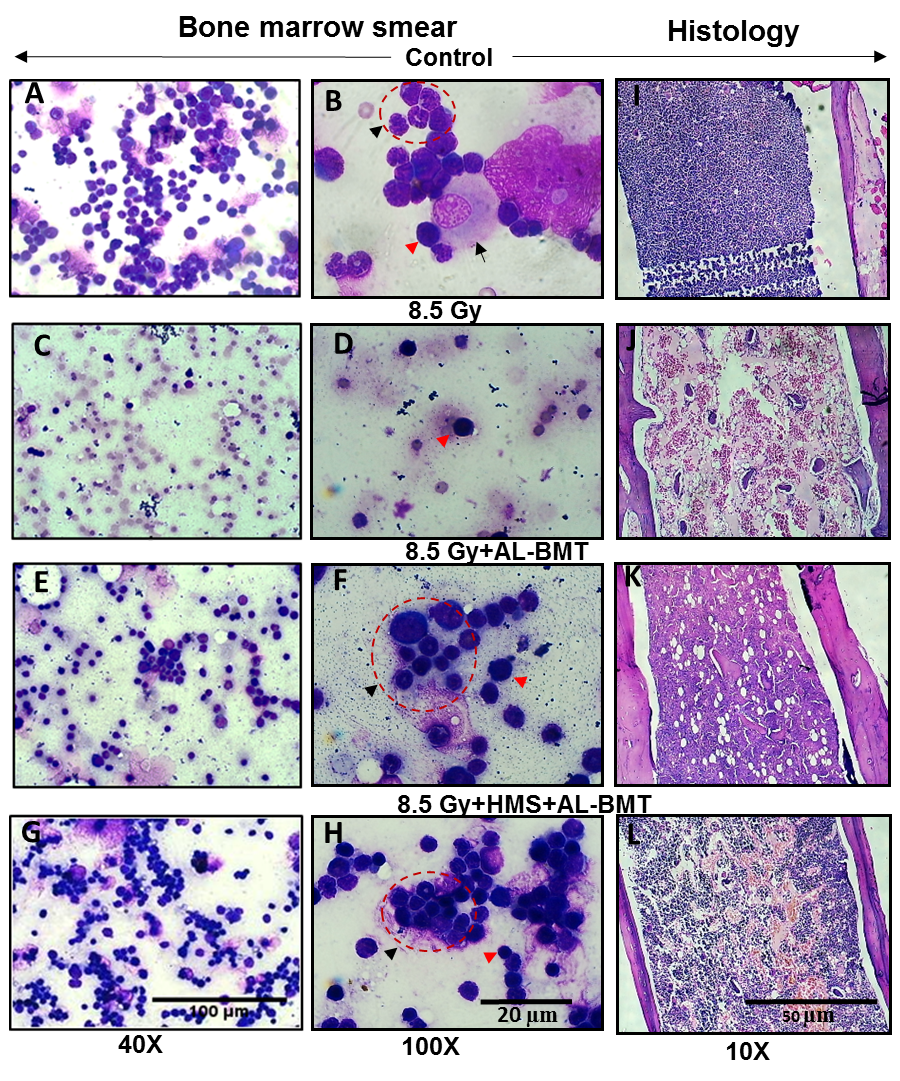


**Supplementary Figure S3**. Autologous BMT accelerates hematopoietic recovery (A-H). Representative images of bone marrow smears prepared 30 days after the mice were transplanted with autologous bone marrow which was collected 2 hours after lethal irradiation (plates E,F) or 8 hours after induction of HMS (plates G,H). Plate’s A,B and C,D represents bone marrow smears prepared from vehicle treated control or irradiated animals on day 30 or 13 respectively. Megakaryocytes, myeloid and erythroid clusters are indicated by black arrow, black and red arrow heads respectively. Plates I-L represent H&E stained femur sections from control mice treated with vehicle, irradiated mice, mice which received AL-BMT and mice which received bone marrow graft collected 8 hours after induction of HMS respectively. Each value is a mean±SEM (n=4-6 animals/group) and comparisons were done as indicated using unpaired t-test. * p<0.05, **p< 0.01, *** p<0.001, ns=not significant).


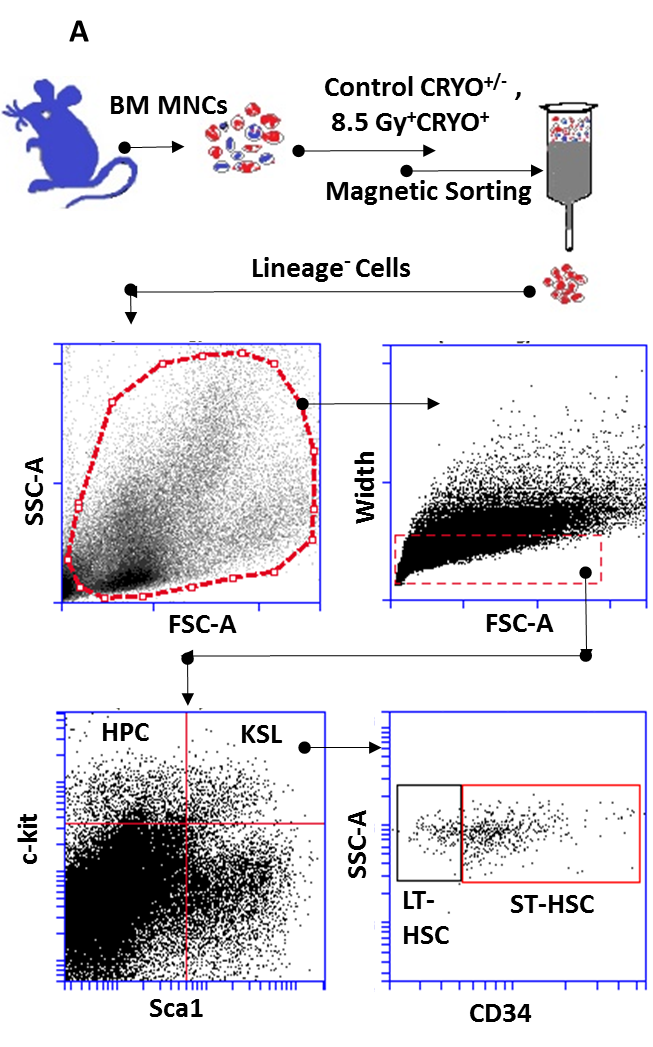


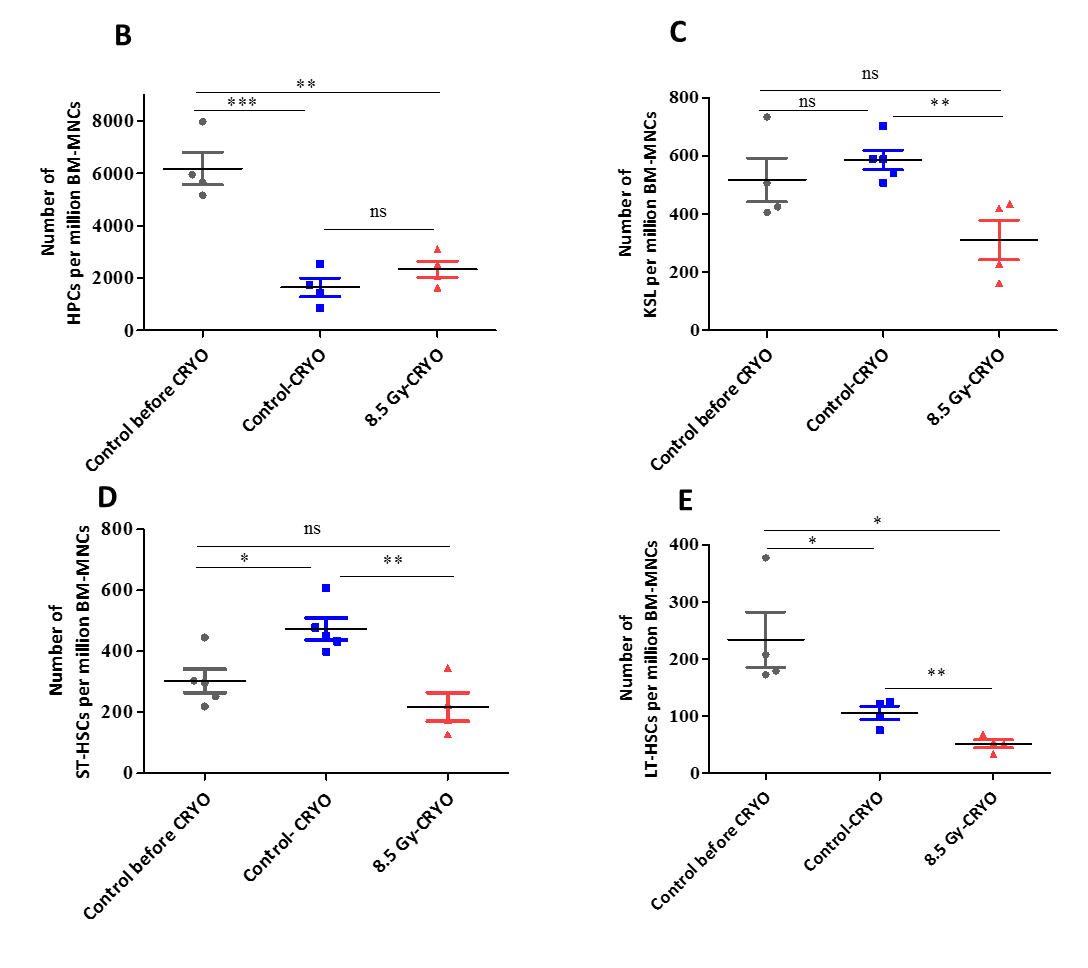


**Supplementary Figure S4**. Quantitative changes in the number of different subsets of HSPCs with indicated treatments. (**A**) Gating strategy for flow cytometric analysis of HPC (Lin^–^Sca1^–^c-kit1^+^ cells), KSL (Lin^-^Sca1^+^c-kit1^+^ cells), ST-HSC (Lin^-^Sca1^+^c-kit1^+^ CD34^+^ cells) and LT-HSC (Lin^-^Sca1^+^c-kit1^+^ CD34^-^ cells) in Lin^-^ fraction of bone marrow samples. Bone marrow grafts collected from vehicle treated un-irradiation control or irradiated animals were either processed immediately or cryopreserved for 22 hours and processed for enumerating HPCs (**B**), KSL (**C**), ST-HSCs (**D**) and LT-HSCs (**E**). Each value is a mean±SEM (n=4-6 animals/group) and comparisons, as indicated, were done for statistical significance using unpaired t-test. * p< 0.05, **p< 0.01, *** p<0.001, ns=not significant.


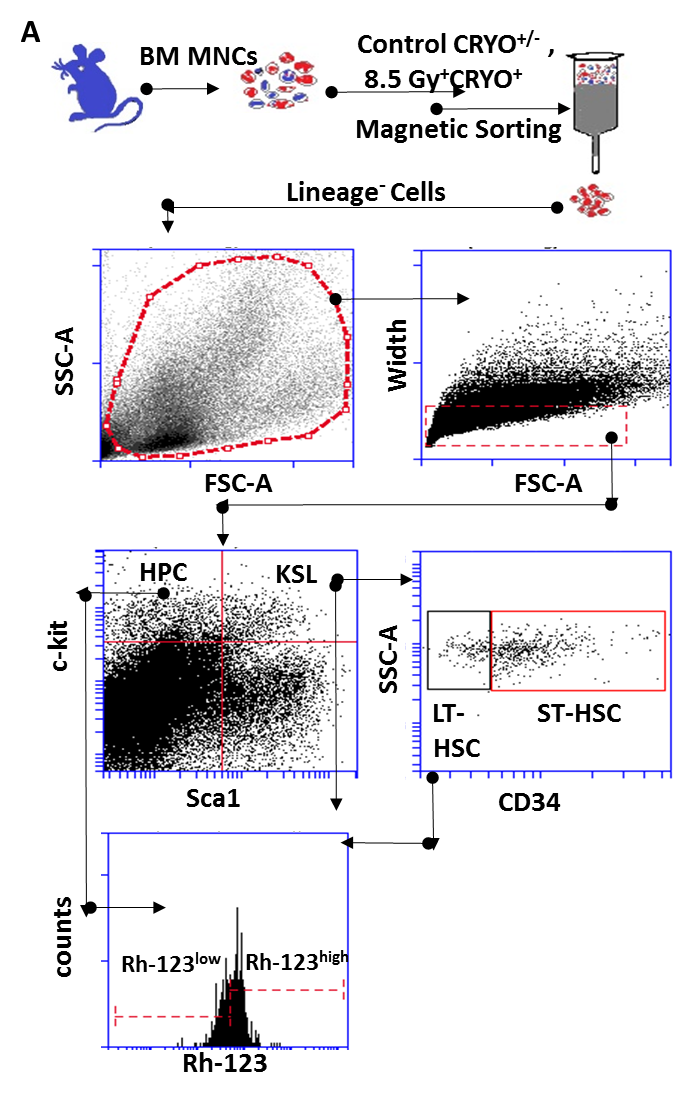


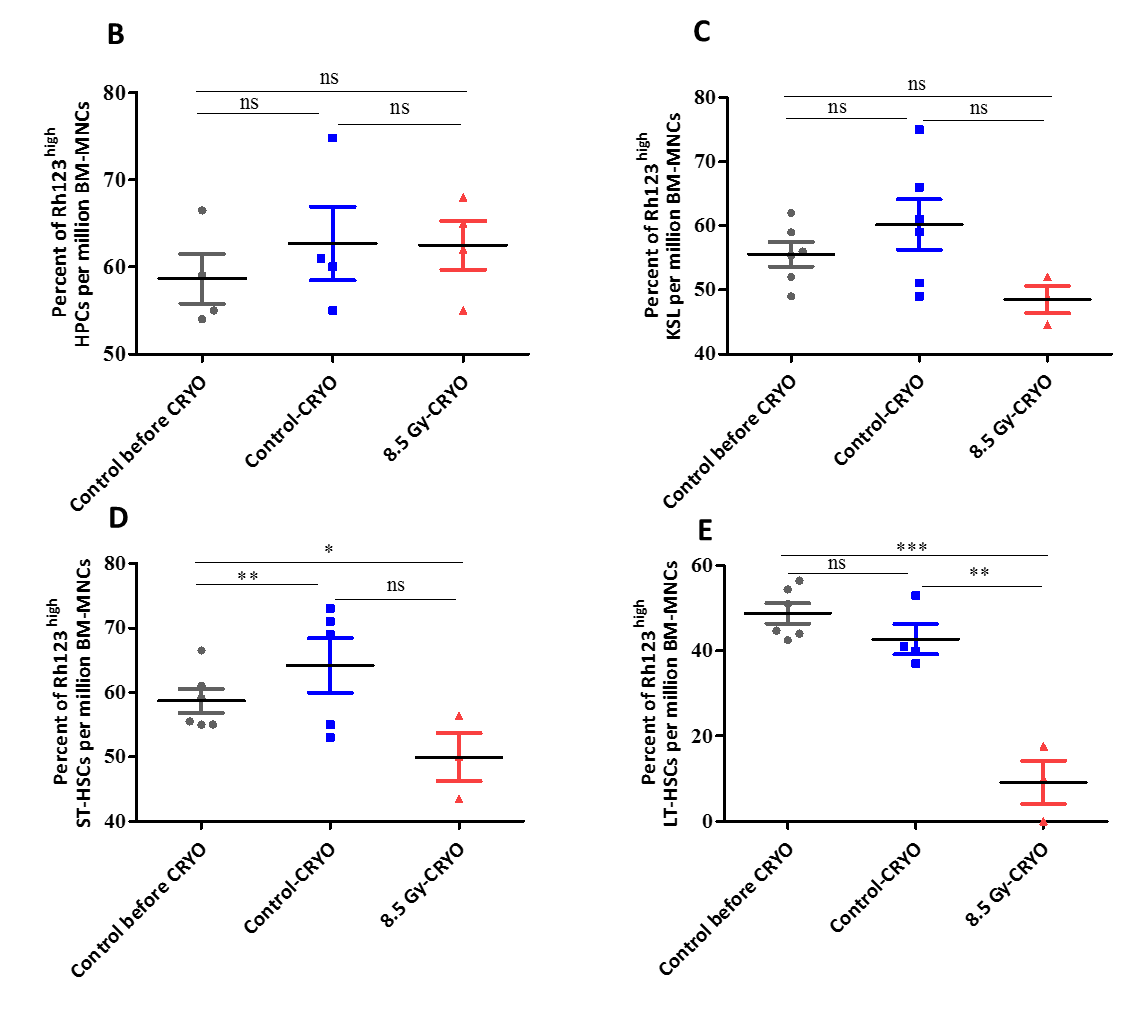


**Supplementary Figure S5**. Effect of cryopreservation on mitochondrial membrane potential in different subsets of HSPCs. (**A**) Gating strategy for flow cytometric analysis of mitochondrial membrane potential in different subsets of HSPCs. Bone marrow samples collected from vehicle treated un-irradiation control or irradiated animals were either processed immediately or cryopreserved for 22 hours and processed for quantifying the fraction of HSPCs with highly polarized mitochondria. (**B**) HPCs, (**C**) KSL, (**D**) ST-HSCs and (**E**) LT-HSCs. Each value is a mean±SEM (n=4-6 animals/group) and comparisons, as indicated, were done for statistical significance using unpaired t test. *p<0.05, **p<0.01, ***p<0.001, ns=not significant.


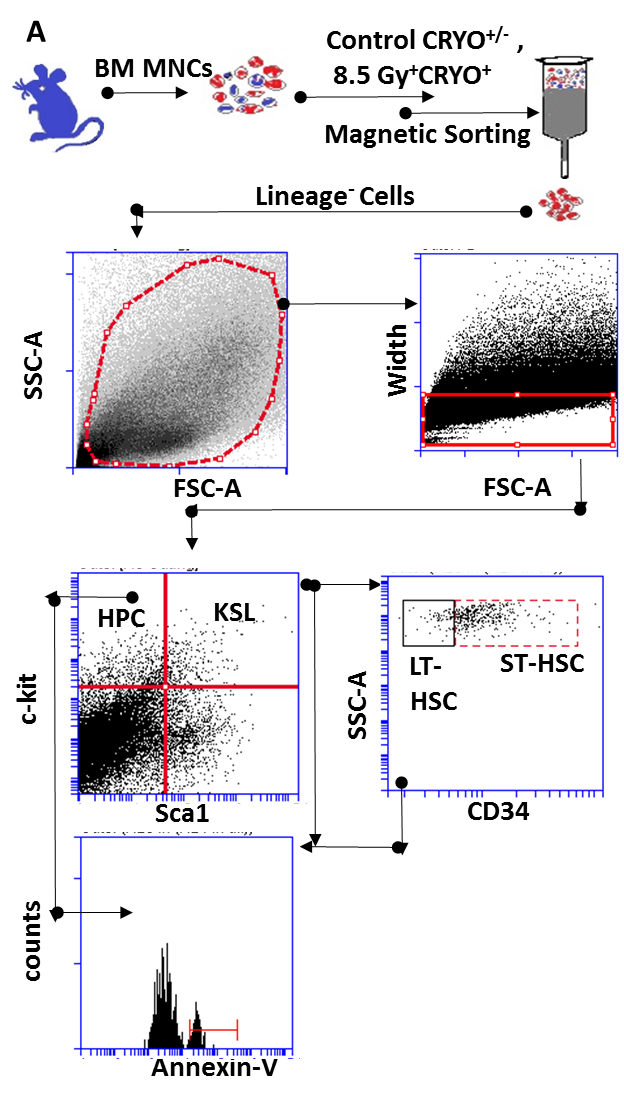


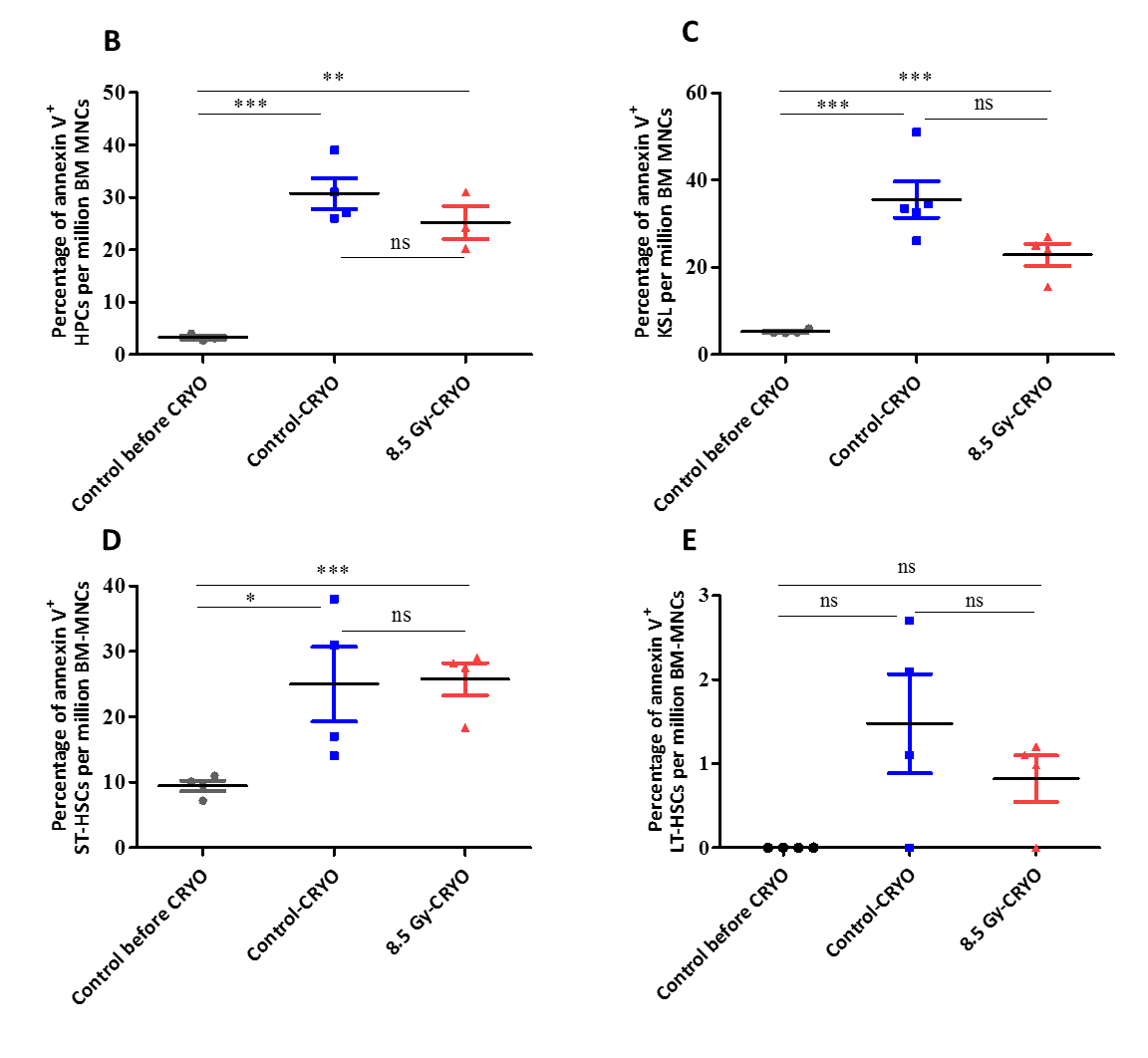


**Supplementary Figure S6**. Effect of cryopreservation on induction of apoptosis in different subsets of HSPCs. The apoptotic fraction (annexin V positive) in different subsets of HSPCs was quantified in bone marrow samples harvested from vehicle treated un-irradiation control or irradiated animals. The samples were either processed immediately or cryopreserved for 22 hours followed by processing for quantification of the apoptotic fraction in different subsets of HSPCs. (**A**) Gating strategy for flow cytometric analysis of annexin V positive fraction in different subsets of HSPCs. (**B**) HPCs, (**C**) KSL, (**D**) ST-HSCs and (**E**) LT-HSCs. Each value is a mean±SEM (n=4-6 animals/group) and comparisons, as indicated, were done for statistical significance using unpaired t test. *p<0.05, **p< 0.01, *** p<0.001, ns=not significant.


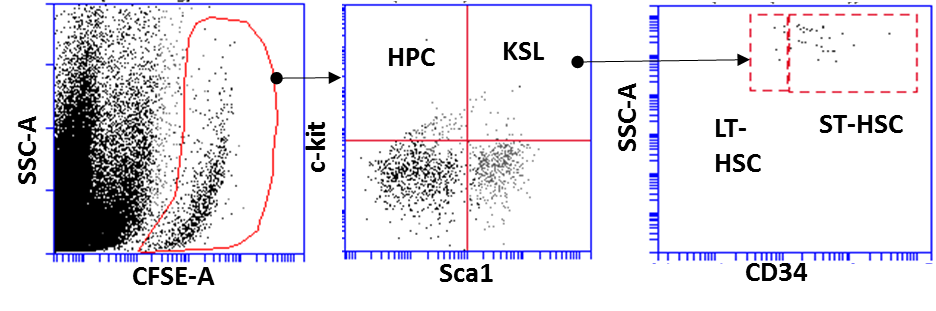


**Supplementary Figure S7**. Homing of different HSPCs in autologous and syngeneic BMT setup. Gating strategy for flow cytometric analysis of homed CFSE^+^ cells in bone marrow 24 hours after transplantation.


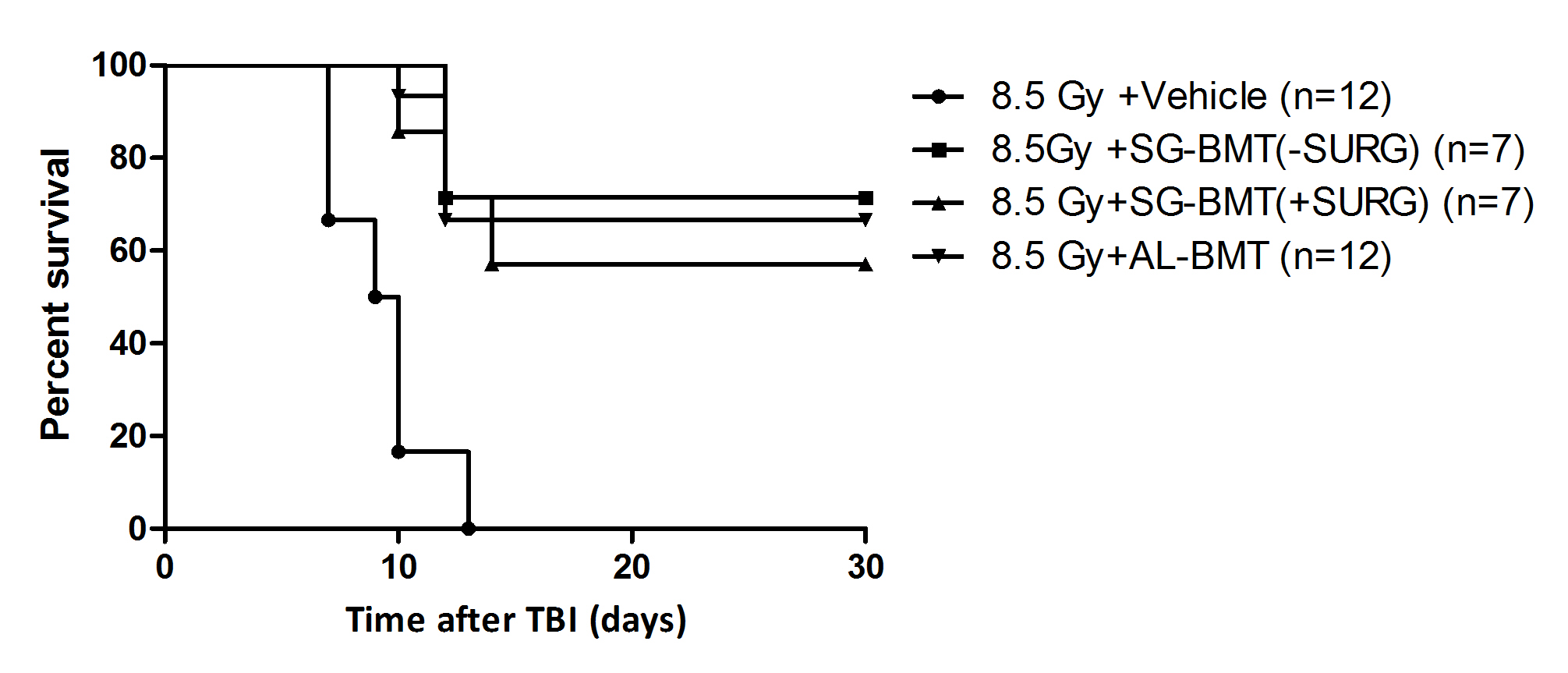


**Supplementary Figure S8**. Surgical stress reduces the HSPCs engraftment and radiomitigative action of both AL-BMT and SG-BMT set ups. Kaplan-Meier plot showing survival rates of mice with different treatments.
